# Disruption of Circadian Sleep/Wake Rhythms in Infants May Herald Future Development of Autism Spectrum Disorder

**DOI:** 10.3390/clockssleep6010012

**Published:** 2024-03-15

**Authors:** Teruhisa Miike, Kentaro Oniki, Makiko Toyoura, Shiro Tonooka, Seiki Tajima, Jun Kinoshita, Junji Saruwatari, Yukuo Konishi

**Affiliations:** 1 Hyogo Rehabilitation Central Hospital, Children’s Sleep and Development Medical Research Center, Kobe 651-2181, Japan; toyoura.makiko@gmail.com (M.T.); seiki.tajima@ohisama-kids-clinic.com (S.T.); 2 Department of Child Development, Kumamoto University, Kumamoto 860-8556, Japan; 3 Division of Pharmacology and Therapeutics, Graduate School of Pharmaceutical Sciences, Kumamoto University, Kumamoto 862-0973, Japan; oniken@kumamoto-u.ac.jp (K.O.); junsaru@gpo.kumamoto-u.ac.jp (J.S.); 4 Kagoshima Comprehensive Clinic for Disabled Children, Kagoshima 891-0175, Japan; shirotono@po5.synapse.ne.jp; 5 Japanese Association of Baby Science Learners, Tokushima 770-0052, Japan; ged.the.archmage@gmail.com; 6 Center for Baby Science, Doshisha University, Kyoto 619-0225, Japan; drkonisi@dream.ocn.ne.jp

**Keywords:** ASD, infants, circadian rhythm, sleep disorder, melatonin‚ prophylactic therapy

## Abstract

We investigated whether the abnormal rhythms in infants are related to the future development of autism spectrum disorder (ASD), using a questionnaire from September to October 2016. The parents of 160 children with ASD (male, n = 123; female, n = 37) were recruited from two hospitals in K and H cities, and as a control group, 145 children (male, n = 75; female, n = 70) were recruited from four nursery schools in T city. The associations between ASD and bedtime and waking time on weekdays and weekends in infancy (<1 years of age), at 1–3 years, and at 3–5 years of ages were studied using a multivariable logistic regression analysis. In particular, at <3 years of age, the following factors were associated with an increased prevalence of ASD in the future: (1) short sleep periods (<8 h); (2) taking a long time to fall asleep (>60 min); (3) sleep beginning after 22:00; (4) a wake-up time after 08:00; and (5) frequent (>3 times) and long-term awakening periods (>60 min). The misalignment and/or shift of the circadian rhythm in infants may be one of the precursors and/or risk factors for the future development of ASD.

## 1. Introduction

The prevalence of autism spectrum disorder (ASD) is considered to be increasing and has become a “serious problem” in modern society [1]; however, the pathogenesis that underlies this condition and the cause(s) of this rapid increase remain unclear. In any case, the early diagnosis and treatment of ASD are desired.

Although some prodromes in ASD have been reported by 6–12 months of age [2,3], no clear symptoms leading to a diagnosis have been confirmed. Thus, the initial diagnosis is generally made at around 1.5–3 years of age. At this time, impaired social communication, which is considered to be one of the main symptoms of ASD, becomes apparent.

However, the abnormal behaviors of individuals with ASD can be recognized even in the newborn period, including infrequent crying or not seeking human interaction or stimulation and/or conversely being intensely irritable and over-reactive to any form of stimulation [4]. Those who perform the former behaviors are called “apathetic-type” or “under-reactive type” neonates, while the others are called “irritable-type” or “over-reactive-type” neonates. 

Although it is less noticeable, sleep disorder should be added as a prodromal symptom in ASD because it is well known to be a major symptom of ASD in infancy/early childhood [5,6], as well as among children and adolescents [7]. Importantly, sleep disorder in ASD is recognized as a circadian rhythm abnormality [8,9,10]. 

In fact, the sleep disorders observed in ASD include increased bedtime resistance, insomnia, awakening, waking difficulties in the morning, and daytime sleepiness, which suggests the existence of circadian rhythm abnormalities [11]. 

In the modern day, the incidence of ASD has increased, so it is important to identify the most influential changes in the living environment that are detrimental to modern humans’ health. Many pathological conditions are known to develop due to the addition of living environmental factors, even those with a genetic background. 

From this viewpoint, it makes the most sense to pay attention to biological clock formation, which controls human life-sustaining functions [12,13]. In addition, it is well recognized that the sleep/wake rhythm is controlled by the biological clock. In the brain, sleep is associated with brain development, growth, damage repair, protection of brain function, and further evolution. However, in modern society, the sleeping environment has changed drastically, and the sleeping environments of children continue to change. For example, in Japan, the most common sleep onset time in infancy was around 20:00 in the 1960s, while at present, 10–30% of children fall asleep after 22:00, which equates to a more than 2 h shift.

Considering the rhythm is quite meaningful when discussing the mental and physical development and health of children [14].

Actually, it has been suggested that, in ASD, biological rhythm disorders are not only associated symptoms but also deeply related to its pathogenesis [15,16,17,18]. More importantly, the circadian rhythm controls the so-called life support systems (e.g., the autonomic nervous system, hormone secretion, and thermoregulation) [13,16]. Therefore, chronobiological theory is logical and convincing for understanding and/or explaining the various clinical manifestations that spread over the whole body of ASD [15,16,19].

According to the principles of early diagnosis and early treatment, we investigated whether the abnormal rhythms in infants are related to the future development of ASD.

In this study, we express a new concept regarding ASD from a chronobiological perspective that has been discussed in recent years [10,11,15,16], and we discuss possible treatment strategies. We hope that this study will provide information that improves our understanding of problems with biological clock formation as an important background factor for sleep disorders in ASD and ASD itself.

## 2. Results

The subjects of this retrospective analysis included 160 children with ASD (Table 1).

**Figure 1:**
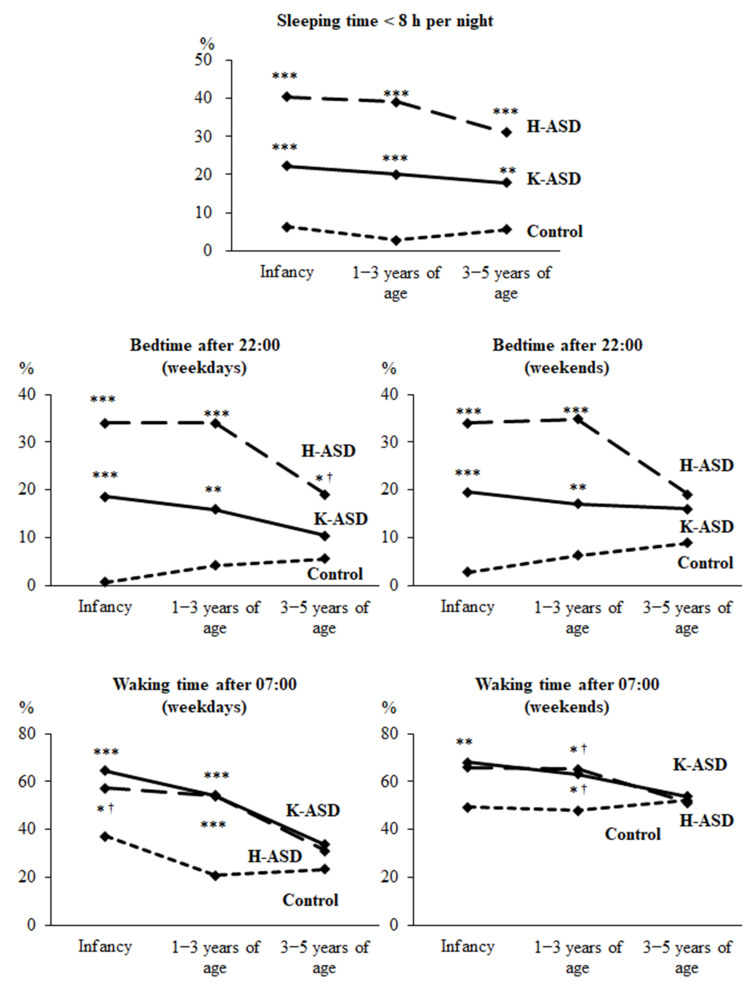
**Sleep status from infancy to 5 years of age in the K-ASD, H-ASD, and control groups.** Chi-squared test (vs. control). * *p* < 0.05, ** *p* < 0.01, *** *p* < 0.001. ^†^ Significance disappeared after Bonferroni correction. Short night sleep (<8 h), sleep onset after 22:00, and wake-up after 7:00 have been shown to be possible risk factors for the future development of ASD, especially up to 3 years of age.

First, we compared the sleeping time <8 h per night, bedtime after 22:00, and waking time after 7:00 between the ASD and control groups (Figure 1). According to Figure 1, in both the ASD groups, the number of incidences of sleeping <8 h per night in infancy, at 1–3 years, and at 3–5 years of age was significantly higher in comparison to that of the control group (Figure 1). In both the ASD groups, the number of incidences of going to bed after 22:00 on weekdays and weekends in infancy and at 1–3 years of age was significantly higher in comparison to that of the control group (Figure 1). In the K-ASD group, but not the H-ASD group, the number of incidences of waking after 7:00 on weekdays and weekends in infancy and at 1–3 years of age was significantly higher in comparison to that of the control group (Figure 1). However, there was no difference between the ASD and control groups in the number of incidences of going to bed after 22:00 and waking after 7:00 at 3–5 years of age (Figure 1).

A total of 113 children with ASD were recruited from the K-Development Support Center for Children (K-ASD), 47 children with ASD from the H-Children’s Sleep and Development Medical Research Center (H-ASD), and 145 control children. Table 1 shows the age and gender of the subjects at the time the questionnaires were distributed and completed.

Next, we analyzed the associations between the prevalence of ASD and bedtime and waking time on weekdays and weekends in infancy, at 1–3 years, and at 3–5 years of age using a multivariable logistic regression analysis (Table 2, Table 3, Table 4 and Table 5). The results of the Hosmer–Lemeshow test using the χ-squared distribution show that the *p* values were greater than 0.05 for all multivariate logistic regression models, confirming the predictive ability of the models. Bedtimes after 22:00 on weekdays and on weekends in infancy and at 1–3 years of age were associated with the prevalence of ASD (Table 2 and Table 3). Moreover, in the H-ASD group only, bedtimes after 22:00 on weekdays and on weekends at 3–5 years of age were associated with the prevalence of ASD (Table 2 and Table 3). Waking times after 8:00 on weekdays and on weekends in infancy and at 1–3 years of age were associated with the prevalence of ASD (Table 4 and Table 5). However, there was no association between waking time at 3–5 years of age and the prevalence of ASD (Table 4 and Table 5).

**Figure 2:**
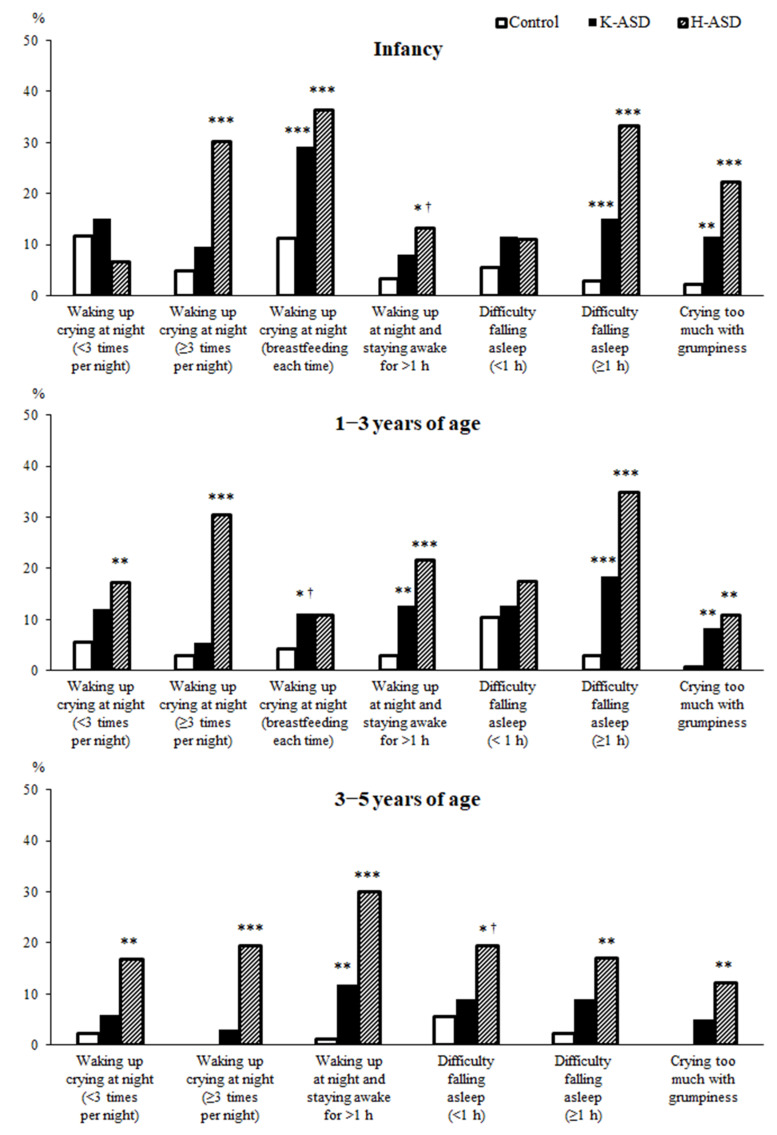
Chi-squared test (vs. control). * *p* < 0.05, ** *p* < 0.01, *** *p* < 0.001. ^†^ Significance disappeared after Bonferroni correction. Long time to fall asleep (>60 min), and frequent (>3 times) and long-term awakening (>60 min) in infancy and early childhood are considered to be possible risk factors for the future development of ASD.

Finally, we analyzed whether there were differences between the children with ASD and the control group regarding the presence or absence of sleep disturbance characteristics from infancy to 5 years of age (Figure 2). During infancy, the frequency of breastfeeding at each time point was significantly higher in both the ASD groups, and the children awoke later. In the H-ASD group, from infancy to 5 years of age, the rate of waking up crying at night three or more times was significantly higher than that in the control group (Table 4). However, in the K-ASD group, although the rate tended to be higher, this difference was not statistically significant (Figure 2). The frequencies of having difficulties falling asleep (≥1 h) and crying too much and being grumpy in infancy and at 1–3 years of age were higher in both the ASD groups than in the control group (Figure 2). The frequencies of waking up at night and staying awake for more than 1 h at 1–3 years and at 3–5 years of age were higher in both the ASD groups than in the control group (Figure 2). Table 6 shows a summary of the results of this study (Table 6).

## 3. Discussion

The results of this study suggest that abnormalities in sleep/wake rhythms, as shown in Table 6, which are common in infancy and early childhood, may be closely associated with the future development of ASD.

Actually, it has been suggested that biological rhythm disorders in ASD, including the sleep/wake rhythm, are not only associated symptoms but also deeply related to the pathogenesis [15,16,17,18]. More importantly, the circadian rhythm’s biological clock controls the so-called life support systems (e.g., the autonomic nervous system, hormone secretion, thermoregulation, energy metabolism, the immune system, coordinated movement, and maintenance of brain function balance [13,16]. Therefore, chronobiological theory is logical and convincing for understanding and/or explaining the various clinical manifestations of ASD [15,16,19], including sleep disorder, epilepsy, enuresis, autonomic disorder, language problems, intellectual disabilities, gastrointestinal symptoms, diabetes, and depression.

However, whether the onset of ASD is a primary matter and sleep disorder is only a secondary concomitant symptom [20] and, conversely, whether sleep disorder appears first as the main cause of ASD development [17,18] have long been matters of debate. In drawing conclusions, we would like to introduce an important report that emphasized that—under all circumstances—children with sleep disorder require quick treatment, and there is recognition of the fact that sleep disorder has a negative effect on children’s growth and development [21,22,23].

From infancy to 5 years of age, some of these sleep problems change slightly and tend to improve with age, while others persist throughout the period. For example, the frequency of night awakenings and daytime crying and grumpiness tends to improve with age. However, short sleep (<8 h at night), awakening at night for >1 h, and the onset of sleep lasting >1 h persist as characteristic symptoms, suggesting that they may serve as predictors of the future development of ASD. The above-mentioned sleep/wake rhythm abnormalities are considered to be characterized by impaired sleep persistence at night and inadequate late sleep onset (in other words, shifted sleep onset) and, as a result, a late awakening time. In other words, the background of these symptoms may be the immaturity and/or misalignment (including shift) of circadian biological clock formation. In fact, circadian rhythm sleep disorder, which also falls within the definition of chrono-disruption [13,24,25], has already been reported before the onset of ASD [17] and even suggested in neonates [4,18].

It has been reported that the expected nighttime sleep duration is approximately 5–6 h at 2 months [26], 8–9 h at 4 months [27], and 8–12 h at 6–7 months [28]. Thus, it has been reported that children who can maintain a good night’s sleep have a high self-soothing ability [27,29] and therefore cry less at night. In order to foster self-soothing habits, it is necessary to improve the response of the guardian who reaches out as soon as the baby cries [30]. These reports suggest that infants who can already sleep through the whole night (8–12 h) from 4–6 months of age do not require night feeding [27]. In addition, our data show that breast feeding during each nighttime awakening during infancy can interfere with sleep and thus contribute to the development of future sleep disorder and possibly ASD itself.

A study of children of 6 months to 4 years of age in Canada [31] reported that most children have a nighttime sleep duration of 10 h (47.6%) or 11 h (42.8%). However, according to our unpublished data from approximately 5000 Japanese children of 2 months to 7 years of age, the study population slept for 9–11 h at night (average of 10 h). We called 9–11 h of sleep the “nighttime basic sleep duration (NBSD),” which we consider to be the duration of sleep required from infancy to 9 years of age.

We have no explanation for the background of short sleep (<8 h). However, when efforts are made to adequately correct the sleep/wake rhythm, the sleep duration sometimes becomes longer than before treatment, which suggests that an inadequate timing system is one of the background factors. These data suggest that, in addition to an individual’s innate nature, short sleep occurs in part due to a night shift in daily life (the so-called “owl type”). In addition to the length of sleep, it is important to sleep continuously throughout the night without waking up. Therefore, it is necessary to pay attention when an individual awakens three or more times at night [32,33] or when they awaken for 20 min (ICSD-3) or more. According to our data, individuals who awaken three or more times or who awaken for more than 60 min should be treated for sleep fragmentation and/or sleep persistence disorder, especially from the latter half of infancy to early childhood.

More importantly and interestingly, individuals with a late bedtime (after 22:00) and a late morning waking time (after 8:00) showed a higher risk ratio for future ASD (refer to the tables and figures). This phenomenon is expressed by difficulty in falling asleep (poor sleep onset), which is significantly observed in children with ASD from infancy to early childhood. The phenomenon indicates a situation in which the function of the biological clock cannot induce sleep in an appropriate time period and can be interpreted as a deviation from the living time zone (shifted biological clock). In addition, we must also give due consideration to the genetic background [34], especially the Clock gene [12]. This delayed sleep onset time is considered to be a so-called delayed sleep phase, and mutation in the period 2 gene was reported as a background factor of this phenomenon in ASD [35]. According to this information, it may be important to know when and how circadian rhythms are formed. The formation of chronobiology is known to be deeply related to the relationship between mother and fetus during pregnancy [36,37]. Importantly, a recent paper reported that the children of mothers who had slept for less than 6 h or longer than 10 h a night were more likely to have ASD. Thus, improving sleep and increasing exercise during pregnancy might reduce the risk of ASD in children [38]. It has also been reported that sleep/wake rhythm abnormalities in neonates, such as sleep onset difficulty, frequent awakening, short sleep, and grumpiness during the day (the so-called “irritable type”), may be the background of the difficulty of raising children, sometimes resulting in abuse and considered to be precursors to the future development of ASD [18].

This information should not be considered a criticism of expectant mothers or the family’s lifestyle, but it should be regarded as information that aims to protect the physical and mental health and well-being of both babies and their family members.

A newborn’s daily life is mainly controlled by the ultradian cycle, with a sleep/wake rhythm of approximately three and/or four hours [39]. In addition, the formation of the chronobiological circadian clock also starts in the fetal period [36] and is thought to be completed at approximately 1 year and 6 months after birth [31,38]. Interestingly, Bruni et al. reported that sleep patterns change during the first year of life but that most sleep variables (i.e., sleep latency and duration) showed little variation from 6 to 12 months. These data provide a context for clinicians to discuss sleep issues with parents and suggest that prevention efforts should focus on the first 3–6 months since sleep patterns show stability from that time point to 12 months [40]. Thus, it can be inferred that it is important to start treatment for circadian rhythm sleep/wake abnormalities in infancy and/or early childhood before the circadian rhythms are completely formed and before the function is fixed. Perhaps this can be considered a prophylactic treatment for ASD, as this is also a time when clinical symptoms (other than the sleep/wake rhythm) are unclear. It is therefore possible to say that providing treatment for sleep disorder in infancy—especially adjusting the sleep/wake circadian rhythm and correcting nocturnal awakening—may have a preventive effect against the future onset of ASD.

Considering that the circadian rhythm biological clock is almost completed after 1 year and 6 months of age, a report recommended that the sleep onset time be set to 19:00 to 20:00 by 1–4 months after birth [28]. This is considered to be important for the formation of an appropriate circadian rhythm. In addition, the formation of an appropriate biological clock will be more suitable for the child’s future school and social life. Natural awakening from 6:00 to 7:00 is an extremely important habit. Since it is necessary to secure the length of the NBSD, the appropriate sleep onset time is 19:00 for children with an NBSD of 11 h and 20:00 for children with an NBSD of 10 h. It has been recommended that night sleep should be from 19:00 to 07:00. We have recommended that—in early infancy—the sleep onset time be set between 19:00 and 21:00. This is because an appropriate sleep/wake habit is useful for ensuring a comfortable daily life and the balanced development of the child [28] and it prevents the future onset of ASD. We would like to emphasize that—based on our experience in daily medical care—adjustment of the sleep/wake rhythm can achieve significant improvements in the core symptoms of ASD.

For children, during infancy, the whole family needs to work together to develop the habit of falling asleep from 19:00 to before 21:00.

It is recommended that, for 10–14 days, the whole family finishes their meals, bathing, and other activities by 19:00 to 20:00; turn off the lights at an appropriate time; and sleep. In many cases, this method works. However, it does not work as well for children with such characteristics as an irritable disposition, which occurs during the neonatal period. Since the response from the newborn period has not yet been established, it makes sense to monitor the progress and correct the daily life rhythm at an appropriate time after entering the infancy period (e.g., 3–6 months). This is based on the idea that the correction of sleep/wake rhythms may be able to properly engage the gears of the biological clock of the whole body.

Since sleeping at night in infants is a transition period from the ultradian rhythm to the circadian rhythm, awakening at a certain time (1–4 h) is physiologically likely to occur. As a response to this awakening and/or false awakening, there is a strong tendency for Japanese guardians to encourage children to fall asleep again by breastfeeding every time. In other words, breastfeeding is often used to deal with midnight awakening in infancy and very early childhood. It is known that children with nighttime feeding habits tend to experience more frequent awakening. Some reports have called attention to the fact that frequent care by parents during the nighttime awakening of children, especially food intake (breast milk, milk, tea, water, etc.), stimulates the peripheral (visceral) clock, causing an arousal reaction and sleep disorder [41,42,43].

For frequent night awakening (>3 times) from 2 months of age, guardians should not respond immediately and instead should watch over the child to help them fall asleep naturally (self-soothing) [29,43,44]. In addition, it has been recommended that night feeding be discontinued after the neonatal period, and/or that the number of feedings be reduced from 2 months and that feeding be discontinued by 3–4 months of age. As mentioned above, it is known that nighttime sleep duration is extended to 8–9 h at 4 months of age, and feeding is not always physically or nutritionally necessary [27,28].

In addition to genetic predisposition and the child-rearing environment, it is also important to review the nighttime habits of modern society, which have an adverse effect on children’s sleeping environments. The International Survey Research on Home Education in Early Childhood, which covered working parents with young children, was carried out in metropolitan areas of four countries (Japan, China, Indonesia, and Finland) in 2018. The result showed that the home-coming time of mothers in Japan and China peaked at 18:00 to 19:00, while that of mothers in Indonesia and Finland peaked at 16:00 to 17:00. The home-coming time of fathers in Japan ranged from 19:00 to 00:00, while that in China peaked at 18:00 and 19:00, that in Indonesia peaked at 19:00 and 20:00, and that in Finland peaked at 16:00 to 17:00. The home-coming time of fathers in Japan was remarkably later in comparison to that of fathers in other countries.

Nowadays, the nocturnal lifestyle habits of modern people who work or remain active late at night under bright lights have become part of the daily routine not only in Japan but also in other countries across the world. The sleep onset time of children involved in such a late-night lifestyle is apt to become late, even close to midnight, especially on weekends. Society (guardians) needs to consider children who grow up in such a poor sleeping environment.

## 4. Materials and Methods

### 4.1. Participants

In this retrospective analysis, we used questionnaires to assess the parents of 113 K-ASD children (male, *n* = 88; female, *n* = 25) recruited from the K-Development Support Center for Children, 47 H-ASD children (male, *n* = 35; female, *n* = 12) recruited from the H-Children’s Sleep and Development Medical Research Center, and 145 children without a diagnosis of ASD (male, n = 75; female, n = 70) recruited from four nursery schools in T city (control). At the time the questionnaires were distributed and completed, the ages of children both with and without ASD ranged from 2 to 6 years. Children taking medications for conditions such as epilepsy were excluded. Children with ASD were recruited from K and H cities, while the children in the control group were recruited from four nursery schools in T city, Japan. There is no time difference between these three cities. The parents of the children with ASD and the children in the control group were asked to complete a questionnaire. This study was conducted in compliance with the Declaration of Helsinki [45] and was approved by the Ethics Committee of C-Hospital (Approval code number 1532, 25/03/2016). All the parents of the children gave their written informed consent before participating in the study. All analyses were conducted in accordance with the Ethical Guidelines for Epidemiological Research in Japan.

### 4.2. Questionnaire Items

A questionnaire was used to investigate the sleep patterns (sleep time, bedtime, and waking time) and the presence or absence of characteristics of a sleep disorder in the children with ASD and in the controls in infancy (<1 years of age), at 1–3 years, and at 3–5 years of ages. The questionnaire investigated the following sleep patterns: (1) sleeping time <8 h per night (yes/no); (2) bedtime on weekdays: before 21:00, 21:00 to 22:00, 22:00 to 23:00, 23:00 to 0:00, or after 0:00; (3) bedtime on weekends: before 21:00, 21:00 to 22:00, 22:00 to 23:00, 23;00 to 0:00, or after 0:00; (4) waking time on weekdays: before 7:00, 7:00 to 8:00, 8:00 to 9:00, or after 9:00; and (5) waking time on weekends before 7:00, 7:00 to 8:00, 8:00 to 9:00, or after 9:00. The characteristics surveyed in the questionnaire to investigate the presence or absence of a sleep disorder were as follows: (1) waking up crying at night (≥3 times per night); (2) waking up crying at night (<3 times per night); (3) waking up crying at night (breastfeeding each time); (4) waking up at night and staying awake for ≥1 h; (5) difficulty falling asleep (<1 h); (6) difficulty falling asleep (≥1 h); and (7) crying too much and being grumpy.

### 4.3. Statistical Analyses

Fisher’s exact test was used for a comparison of categorical variables. The associations between the prevalence of ASD and bedtime and waking time on weekdays and weekends in infancy, at 1–3 years, and 3–5 years of ages were studied using a multivariable logistic regression analysis. These associations were measured using the odds ratio (OR) and 95% confidence interval (95% CI) for the prevalence of ASD. In the multivariable logistic regression model, the ORs were adjusted by adding sex as an explanatory variable. A Hosmer–Lemeshow test was performed using an χ-squared distribution to confirm the predictive value of the multivariable logistic regression models. *p* values of <0.05 were considered to be statistically significant. Multiple comparisons were corrected using the Bonferroni method, and *p* values of <0.05/n were considered statistically significant after correcting for the number of comparisons (n) made.

## 5. Conclusions

The present study suggests that abnormalities in the sleep/wake rhythm seen from infancy to early childhood may be closely related to the onset of sleep disorders in the future and the onset of ASD. As a prophylactic therapy against the onset of sleep disturbances in ASD and/or the clinical onset of ASD itself, it is recommended to make efforts to fix the abnormal sleep/wake rhythms before circadian rhythms are fully formed (3–18 months) and anchored in the suprachiasmatic nucleus.

## Figures and Tables

**Table 1:** Characteristics of the subjects at the time of questionnaire distribution and completion.

	Control		K-ASD		H-ASD	
Age (Years Old)	Male	Female	Male	Female	Male	Female
2	20 (52.6%)	18 (47.4%)	3 (100.0%)	0 (0.0%)	3 (100%)	0 (0.0%)
3	20 (47.6%)	22 (52.4%)	14 (82.4%)	3 (17.6%)	4 (66.7%)	2 (33.3%)
4	17 (50.0%)	17 (50.0%)	24 (75.0%)	8 (25.0%)	16 (84.2%)	3 (15.8%)
5	13 (59.1%)	9 (40.9%)	30 (73.2%)	11 (26.8%)	5 (50.0%)	5 (50.0%)
6	5 (55.6%)	4 (44.4%)	17 (85.0%)	3 (15.0%)	7 (77.8%)	2 (22.2%)
Total	75 (51.7)	70 (48.3)	88 (77.8%)	25 (22.1%)	35 (74.5%)	12 (25.5%)
Overall total	145		113		47	

**Table 2:** Associations between bedtime on weekdays or weekends from infancy to 5 years of age and the prevalence of ASD in K-ASD and control children.

Weekdays					Weekends				
	Control	K-ASD	OR (95% CI)	*p*		Control	K-ASD	OR (95% CI)	*p*
Infancy					Infancy				
before 21:00	105	57	1		before 21:00	94	56	1	
21:00 to 22:00	39	35	1.86 (1.04–3.36)	0.037	21:00 to 22:00	47	35	1.41 (0.79–2.49)	0.241
after 22:00	1	21	36.87 (4.74–286.59)	<0.001	after 22:00	4	22	9.17 (2.92–28.81)	<0.001
1–3 years of age					1–3 years of age				
before 21:00	74	44	1		before 21:00	66	42	1	
21:00 to 22:00	62	51	1.42 (0.82–2.45)	0.211	21:00 to 22:00	68	51	1.17 (0.67–2.03)	0.579
after 22:00	6	18	5.90 (2.08–16.76)	<0.001	after 22:00	9	19	3.78 (1.50–9.54)	0.005
3–5 years of age					3–5 years of age				
before 21:00	41	40	1		before 21:00	35	36	1	
21:00 to 22:00	43	55	1.31 (0.71–2.42)	0.396	21:00 to 22:00	47	53	1.15 (0.61–2.19)	0.658
after 22:00	5	11	2.28 (0.69–7.52)	0.175	after 22:00	8	17	2.04 (0.75–5.54)	0.165

Multivariable logistic regression analysis. ORs were adjusted by sex. In all multivariable logistic regression models, the *p*-values for the Hosmer–Lemeshow test were greater than 0.05. OR, odds ratio; CI, confidence interval.

**Table 3:** Associations between bedtime on weekdays or weekends from infancy to 5 years of age and the prevalence of ASD in H-ASD and control children.

Weekdays					Weekends				
	Control	H-ASD	OR (95% CI)	*p*		Control	H-ASD	OR (95% CI)	*p*
Infancy					Infancy				
before 21:00	105	24	1		before 21:00	94	21	1	
21:00 to 22:00	39	7	0.92 (0.36–2.37)	0.867	21:00 to 22:00	47	10	1.13 (0.48–2.65)	0.788
after 22:00	1	16	86.75 (10.40–723.59)	<0.001	after 22:00	4	16	22.88 (6.43–81.51)	<0.001
1–3 years of age					1–3 years of age				
before 21:00	74	18	1		before 21:00	66	16	1	
21:00 to 22:00	62	13	0.84 (0.37–1.89)	0.672	21:00 to 22:00	68	14	0.80 (0.35–1.81)	0.589
after 22:00	6	16	18.52 (5.43–63.20)	<0.001	after 22:00	9	16	12.60 (4.00–39.70)	<0.001
3-5 years of age					3–5 years of age				
before 21:00	41	21	1		before 21:00	35	16	1	
21:00 to 22:00	43	13	0.57 (0.25–1.33)	0.193	21:00 to 22:00	47	18	0.82 (0.36–1.88)	0.639
after 22:00	5	8	5.53 (1.40–21.85)	0.015	after 22:00	8	8	3.55 (1.01–12.50)	0.049

Multivariable logistic regression analysis. ORs were adjusted by sex. In all multivariable logistic regression models, the *p*-values for the Hosmer–Lemeshow test were greater than 0.05.

**Table 4:** Associations between waking time on weekdays or weekends from infancy to 5 years of age and the prevalence of ASD in K-ASD and control children.

Weekdays					Weekends				
	Control	K-ASD	OR (95% CI)	*p*		Control	K-ASD	OR (95% CI)	*p*
Infancy					Infancy				
before 07:00	91	40	1		before 07:00	73	36	1	
07:00 to 08:00	51	46	2.16 (1.22–3.84)	0.008	07:00 to 08:00	65	49	1.78 (1.00–3.15)	0.049
after 08:00	3	27	27.19 (7.37–100.35)	<0.001	after 08:00	6	28	10.84 (3.95–29.75)	<0.001
1–3 years of age					1–3 years of age				
before 07:00	114	52	1		before 07:00	75	41	1	
07:00 to 08:00	28	42	3.88 (2.08–7.25)	<0.001	07:00 to 08:00	63	45	1.49 (0.85–2.63)	0.168
after 08:00	2	19	26.62 (5.66–125.13)	<0.001	after 08:00	6	25	9.67 (3.47–26.97)	<0.001
3–5 years of age					3–5 years of age				
before 07:00	69	69	1		before 07:00	43	48	1	
07:00 to 08:00	20	30	1.66 (0.83–3.32)	0.155	07:00 to 08:00	43	44	0.97 (0.53–1.80)	0.931
after 08:00	1	5	4.38 (0.47–40.83)	0.195	after 08:00	4	12	2.62 (0.75–9.14)	0.132

Multivariable logistic regression analysis. ORs were adjusted by sex. In all multivariable logistic regression models, the *p*-values for the Hosmer–Lemeshow test were greater than 0.05.

**Table 5:** Associations between waking time on weekdays or weekends from infancy to 5 years of age and the prevalence of ASD in H-ASD and control children.

Weekdays					Weekends				
	Control	H-ASD	OR (95% CI)	*p*		Control	H-ASD	OR (95% CI)	*p*
Infancy					Infancy				
before 07:00	91	20	1		before 07:00	73	16	1	
07:00 to 08:00	51	13	1.20 (0.54–2.68)	0.649	07:00 to 08:00	65	16	1.25 (0.57–2.76)	0.575
after 08:00	3	14	30.80 (7.28–130.26)	<0.001	after 08:00	6	15	13.27(4.24–41.61)	<0.001
1–3 years of age					1–3 years of age				
before 07:00	114	21	1		before 07:00	75	16	1	
07:00 to 08:00	28	14	2.91 (1.28–6.63)	0.011	07:00 to 08:00	63	16	1.27 (0.58–2.81)	0.549
after 08:00	2	11	37.43 (7.17–195.51)	<0.001	after 08:00	6	14	14.74 (4.48–48.47)	<0.001
3–5 years of age					3–5 years of age				
before 07:00	69	29	1		before 07:00	43	19	1	
07:00 to 08:00	20	11	1.35 (0.56–3.28)	0.504	07:00 to 08:00	43	15	0.79 (0.35–1.79)	0.571
after 08:00	1	2	10.59 (0.85–131.34)	0.066	after 08:00	4	5	3.13 (0.71–13.74)	0.130

Multivariable logistic regression analysis. ORs were adjusted by sex. In all multivariable logistic regression models, the *p*-values for the Hosmer–Lemeshow test were greater than 0.05.

**Table 6:** Sleep characteristics during infancy to 5 years of age in ASD children.

	Short Sleep (<8 h)	Sleep Onset Time after 22:00, Weekdays	Wake-up Time after 7:00, Weekdays	Waking up Crying at Night (Breast Feeding Each Time)	Waking Up Crying at Night (<3 Times per Night)	Waking up Crying at Night (≥3 Times per Night)	Waking up at Night and Staying Awake for >1 h	Difficulty Falling Asleep (>1 h)	Crying too Much with Grumpiness
Infancy									
K-ASD	***	***	***	***				***	**
H-ASD	***	***		***		***		***	***
1–3 years									
K-ASD	***	**	***				**	***	**
H-ASD	***	***	***		**	***	***	***	**
3–5 years									
K-ASD	**						***		
H-ASD	***				**	***	***	**	**

Chi-squared test (vs. control). ** *p* < 0.01, *** *p* < 0.001.

## Data Availability

The data presented in this study are available on request from the corresponding author due to privacy.

## Abbreviations

ASD	autism spectrum disorder
K-ASD	Kagoshima-Development Support Center for Children-ASD
H-ASD	Hyougo-Children’s Sleep and Development Medical Research Center-ASD
NBSD	nighttime basic sleep duration
